# Viral load non-suppression among adolescents and youth living with HIV in South Africa

**DOI:** 10.4102/sajid.v39i1.629

**Published:** 2024-09-25

**Authors:** Lesiba O. Molopa, Thembelihle P. Ginyana, Noloyiso Vondo, Rindidzani Magobo, Goitseone Maseko, Nompumelelo Zungu, Khangelani Zuma, Leickness Simbayi, Musawenkosi Mabaso, Sizulu Moyo

**Affiliations:** 1 Division of Public Health Societies and Belonging, Human Sciences Research Council, Cape Town, South Africa; 2 Division of Public Health Societies and Belonging, Human Sciences Research Council, Pretoria, South Africa; 3 School of Nursing and Public Health, College of Health Sciences, University of KwaZulu-Natal, Durban, South Africa; 4 School of Public Health, Faculty of Health Sciences, University of the Witwatersrand, Johannesburg, South Africa; 5 Department of Psychiatry and Mental Health, Faculty of Health Sciences, University of Cape Town, Cape Town, South Africa; 6 Division of Public Health Societies and Belonging, Human Sciences Research Council, Durban, South Africa; 7 School of Public Health and Family Medicine, Faculty of Health Sciences, University of Cape Town, Cape Town, South Africa

**Keywords:** HIV, antiretroviral treatment, viral load, non-suppression, adolescent, youth, South Africa

## Abstract

**Background:**

Despite the increased initiation and uptake of antiretroviral therapy (ART) in South Africa, some people living with HIV (PLHIV) who are on ART still have non-suppressed viral load (VL).

**Objectives:**

This study aimed to determine the prevalence of VL non-suppression among adolescents and youth (aged 12 years – 24 years) living with HIV and on ART in South Africa, as well as the factors associated with it.

**Method:**

Data from the 2017 South African national HIV prevalence, incidence, behaviour, and communication survey were analysed. The survey used a multistage-stratified cluster sampling design. A backward stepwise multivariable generalised linear model was used to identify factors associated with VL non-suppression.

**Results:**

The study included 340 participants aged 12 years – 24 years, with a median age of 21 (interquartile range [IQR]: 18–23). The proportion of adolescents and youth living with HIV and on ART with non-suppressed VL was 19.2% (95% confidence interval [CI]: 14.4–25.3). Approximately 60% of the participants were not on ART. The odds of VL non-suppression were significantly higher among youth aged 15 years – 19 years (adjusted odds ratio [AOR] = 1.63 [95% CI: 1.24–2.13], *p* = 0.001) and aged 20 years – 24 years (AOR = 1.22 [95% CI: 1.06–1.41], *p* = 0.005) compared to adolescents aged 12 years – 14 years. The odds were significantly lower among individuals of other races (AOR = 0.80 [95% CI: 0.69–0.92], *p* = 0.003) compared to black African people.

**Conclusion:**

Findings suggest a need for ART education and counselling as part of treatment support. In addition, the promotion of HIV awareness as part of strengthening the HIV treatment and prevention cascade.

**Contribution:**

The article showed the prevalence of VL non-suppression and associated factors among adolescents and youth.

## Introduction

South Africa has the highest burden of HIV globally.^1^ The HIV epidemic affects people of all age groups. In 2022, an estimated 7.6 million people were living with HIV (PLHIV) in South Africa. Of these, 36 000 were newly infected children and adolescents aged 0 years – 19 years and 56 000 were newly infected adolescents and youth aged 15 years – 24 years.^2^ According to the United Nations Population Fund (UNFPA), persons aged 10 years – 19 years are considered adolescents, while persons aged 20 years – 24 years are considered youth.^3^ In high HIV prevalent low and middle income countries such as South Africa, programmatic monitoring of vertical versus horizontal transmission and viral suppression is critical in the national HIV response especially among adolescents and youth.^4,5^ This is because adolescents and youth face unique treatment and care challenges, which vary based on the manner they acquired HIV.^4^

Evidence shows that although there has been a substantial decline in HIV incidence and mortality in children under 14 years of age over the past decades, the epidemic remains a public health challenge and a national priority in South Africa.^2,6^ The extent of viral load (VL) suppression among children and adolescents (0 years – 19 years) has changed little compared to older age groups between 2014 and 2020.^5^ Using data from 2005 to 2019, another study found that the prevalence of VL suppression (< 400 copies/mL) was 72.4% in children aged 0 years – 19 years and 85.7% in adults aged 15 years and older, indicating that younger people are less suppressed than adults.^5^ In 2017, the prevalence of VL suppression (< 50 copies/mL) was 47.2% among adolescents (10 years – 19 years), increasing to 50.5% in 2020.^5^

The decline in HIV incidence and mortality among children can be attributed to several interventions including early testing and improved access to antiretroviral therapy (ART).^6,8^ Successful prevention of vertical transmission of HIV from mothers to their babies either during pregnancy, at delivery, or through breastfeeding, has been cited as a major reason for the drastic reduction in paediatric HIV burden in the country.^6,9^ Among adolescents, efforts to accelerate progress towards the Sustainable Development Goals has resulted in health education, increased condom usage, the provision of incentivised interventions specifically among adolescent girls and the use of pre-exposure prophylaxis.^10,11,12^ Consequently, South Africa has the world’s largest ART programme, with over 5.6 million PLHIV receiving ART in 2022, of whom 123 332 were children aged 0 years – 14 years and 5 574 940 were adolescents and adults aged 15 years and older.^13^

Despite the scaling up of ART and more adolescents and youth receiving ART, VL non-suppression occurs in this population group.^14^ This poses a challenge to achieving the Joint United Nations Programme on HIV and AIDS (UNAIDS) 95-95-95 targets, which call for 95% of all PLHIV to know their HIV status, 95% of all people with diagnosed HIV to receive sustained ART, and 95% of all people on ART to have viral suppression.^15^ All three targets are key to ending the epidemic because HIV positive people on ART with undetectable VL do not transmit HIV through sex with HIV-negative individuals.^16^

The public health and individual benefits of the UNAIDS targets ultimately depend on achieving the third 95 across regions and/or populations. Therefore, it is important to better understand the factors driving VL non-suppression among children and adolescents in South Africa. Evidence shows that poor adherence to treatment and disengagement from care leads to VL non-suppression.^17,18,19^ Poor ART adherence and VL non-suppression are linked to the contextual factors associated with socio-demographic and socio-behavioural characteristics in specific settings.^20,21,22^

This study aims to determine the prevalence and factors associated with VL non-suppression among adolescents and youth (12 years – 24 years) living with HIV and on ART in South Africa.

## Research methods and design

### Data source and study sampling

This secondary analysis used data from the 2017 South African National HIV Prevalence, Incidence, Behaviour, and Communication Survey collected using a complex multistage-stratified randomised cluster sampling design.^23^ Briefly, the survey used a master sampling frame consisting of 15 visiting points or households drawn from 1000 randomly selected small layer areas sampled from 86 000 such areas from the 2001 census updated in 2011.^24^ The selected small areas layers were stratified by province, locality type (urban areas, rural informal or farm areas, and formal or tribal areas), race group, and sex.

### Study procedures

Persons of all ages living in selected households were eligible for the survey. Detailed household questionnaires and age-appropriate individual questionnaires were administered to consenting or assenting individuals with parents and guardians answering on behalf of children under the age of 12 years. The questionnaires solicited information on demographic characteristics and HIV-related knowledge, attitudes, practices, and behaviours.

Dried blood spot cards were used to collect blood specimens from consenting individuals through a finger prick or heel prick in infants. These dried blood spot specimens were tested to determine, among others HIV serostatus, ART exposure (nevirapine, efavirenz, lopinavir, atazanavir and darunavir), and viral suppression. Specimens were sent to a centralised laboratory for HIV antibody testing using a three-step enzyme immunoassay (EIA) algorithm. Samples that tested positive for HIV during the first two EIAs (Roche Elecys HIV Ag/Ab assay, Roche Diagnostics, Manheim, Germany, and Genescreen Ultra HIV Ag/Ab assay, Bio-Rad Laboratories, California, USA) underwent a nucleic acid amplification test (COBAS AmpliPrep/Cobas Taqman HIV-1 Qualitative Test, v2.0, Roche Molecular Systems, New Jersey, USA) for final test result interpretation. Exposure to antiretroviral drugs in HIV positive specimens was determined using high-performance liquid chromatography (HPLC) coupled with tandem mass spectometry. For VL testing, the lower limit for detection was 700 copies/mL – 800 copies/mL. This study used a sub-sample of adolescents and youth aged 12 years – 24 years who were tested for HIV and on ART.

### Measures

#### Dependent variable

The primary outcome was VL non-suppression among adolescents and youth who tested positive for HIV, with a VL ≥ 1000 copies/mL. In the analysis, the outcome is binary where 1 = VL ≥ 1000 HIV RNA copies/mL and 0 = VL < 1000 HIV RNA copies/mL. The cut off is the standard used in the Population-based HIV Impact Assessments,^25^ and the UNAIDS Undetectable = Untransmittable (U = U) definition,^26^ which aligns with international standards.

#### Independent variables

Explanatory variables included age-group in years (12–14, 15–19, and 20–24), sex (male and female), race (black African people and other – white, mixed-race or Asian people), educational level (primary, secondary, and tertiary), locality type (urban areas, rural informal or tribal areas, and rural formal or farm areas) and socio-behavioural variables such as ever had sex in the last 12 months (yes and no), and self-rated health (excellent or good, and fair or poor).

### Data analysis

All analyses were conducted in STATA version 15.1 (Stata Corporation, College Station, Texas, USA) using weighting to adjust for the complex multilevel unequal sampling probabilities in the survey design. Summary statistics were used to describe the study sample and VL non-suppression. A multivariable generalised linear model (GLM) with a backward stepwise selection method was fitted to determine factors associated with VL non-suppression. Adjusted odds ratio (AOR) with 95% confidence intervals (CI) and *p*-values less than 0.05 were used to determine the direction and the strength of the association.

### Ethical considerations

Ethics approval to conduct the study was granted by the Human Sciences Research Council (HSRC) Research Ethics Committee (REC: 4/18/11/15), and the Division of Global HIV and TB (DGHT) and the Centre for Global Health (CHG) of the Centers for Disease Control and Prevention (CDC). Written and verbal informed consent was obtained from all individual participants involved in the study.

## Results

**FIGURE 1:**
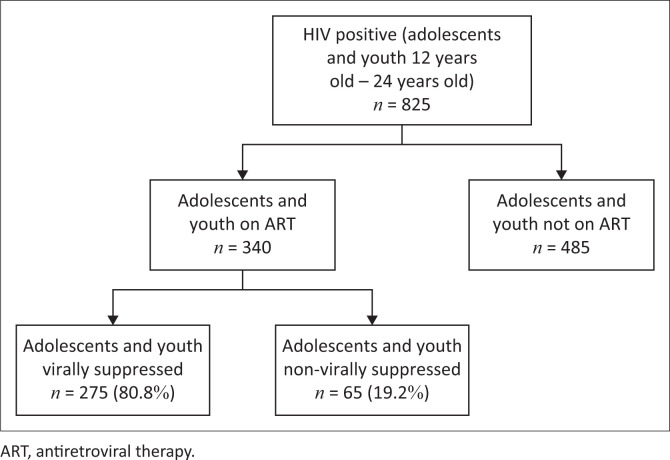
Selection of children and youth included in the study.

Figure 1 shows that of the 825 HIV positive adolescents and youth in the study sample, 340 (41.2%) were on ART. Almost 60% were not on ART despite living with HIV.

### Sample characteristics

Table 1 shows the characteristics of the study sample (*n* = 340), the median age and interquartile range (IQR) were 21 (IQR: 18–23). Most participants were aged 20 years – 24 years old (62%), female (68%), had a secondary-level education (87%), and almost 50% lived in rural informal or tribal areas. The majority reported having engaged in sexual activity in the last 12 months (79%) and rated their health as poor or fair (66%).

**TABLE 1:** Characteristics of HIV positive participants aged 12 years – 24 years on antiretroviral therapy (*n* = 340), South Africa, 2017.

Variables	*n* †	%
**Age in years**		
12–14	23	7.8
15–19	105	30.1
20–24	212	62.1
**Sex**		
Male	84	30.9
Female	256	68.1
**Race groups**		
Black African people	329	96.4
Other	11	3.6
**Education level**		
No education or primary	19	8.4
Secondary	141	87.4
Tertiary	10	4.2
**Locality type**		
Urban areas	126	45.7
Rural informal or tribal areas	174	46.6
Rural formal or farm areas	40	7.7
**Had sex in the last 12 months**		
Yes	168	79.3
No	38	20.7
**Self-rated health**		
Excellent or good	95	34.4
Fair or poor	177	65.6

† , Totals do not all add up to the study sample because of non-response or missing data.

### Viral load non-suppression prevalence and sample characteristics

Table 2 shows the prevalence of VL non-suppression by sample characteristics. Viral load non-suppression was significantly higher among those who reported having had sex in the last 12 months. Although not statistically significant, the prevalence of VL non-suppression was also higher among adolescents aged 12 years – 14 years, males, other race groups, those with no education or only primary education, those who reside in rural informal or tribal areas, and those who reported fair or poor self-rated health.

**TABLE 2:** Viral load non-suppression by sample characteristics among adolescents and youth aged 12 years – 24 years who tested HIV positive and were on antiretroviral therapy (*n* = 340), South Africa, 2017.

Variables	*n* †	%‡	95% CI	*p*
**Age in years**				0.211
12–14	23	35.8	14.9–64.1	-
15–19	105	20.5	12.6–31.5	-
20–24	212	16.6	10.9–24.3	-
**Sex**				0.544
Male	84	21.9	13.2–34.1	-
Female	256	18.1	12.5–25.5	-
**Race groups**				0.893
Black African people	329	19.2	14.2–25.4	-
Other	11	21.0	4.9–57.8	-
**Educational level**				0.410
No education or primary	19	28.6	8.7–62.8	-
Secondary	141	15.2	8.6–25.5	-
Tertiary	10	28.4	7.1–67.5	-
**Locality type**				0.643
Urban areas	126	16.4	10.0–25.8	-
Rural informal or tribal areas	174	21.8	14.7–31.0	-
Rural formal or farm areas	40	20.5	7.2–46.2	-
**Had sex in the last 12 months**				0.030
Yes	168	21.3	13.8–31.3	-
No	38	7.6	2.9–18.4	-
**Self-rated health**				0.231
Excellent or good	95	15.8	8.9–26.5	-
Fair or poor	177	19.4	12.8–28.3	-

CI, confidence interval.

† , totals do not all add up to the study sample because of non-response/missing data.

‡ , row proportions of viral load non-suppression relative to suppressed viral load.

### Factors associated with viral load non-suppression

**FIGURE 2:**
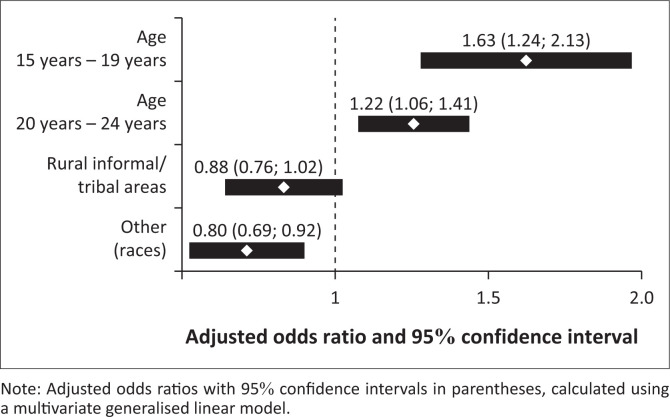
Coefficient plot of the final model for factors associated with viral load non-suppression among adolescents and youth aged 12 years – 24 years who tested positive for HIV and on antiretroviral therapy.

Figure 2 shows the determinants of VL non-suppression in adolescents and youth aged 12 years – 24 years who tested HIV positive and were on ART. The odds of VL non-suppression were significantly higher among the youth aged 15 years – 19 years (AOR = 1.63 [95% CI: 1.24–2.13] *p* = 0.001) and the youth aged 20 years – 24 years (AOR = 1.22 [95% CI: 1.06–1.41] *p* = 0.005) compared to adolescents aged 12 years – 14 years. The odds of VL non-suppression were significantly lower among other races (AOR = 0.80 [95% CI: 0.69–0.92], *p* = 0.003) compared to black African people.

## Discussion

This nationally representative study revealed two very disturbing findings. Firstly, almost 60% of adolescents and youth living with HIV were not on ART, possibly undiagnosed. Secondly and the main outcome of our study, was that almost a fifth of adolescents and youth on ART were not virally suppressed. This is lower than the VL non-suppression observed in the adult population.^22^ Similar observations were made in other Eastern and Southern African countries such as Malawi, Kenya, Uganda, and Zimbabwe.^18,27,28,29^ This remains a concern given the ongoing efforts to end the epidemic in the country, and the burden of HIV among youth especially females aged 15 years – 24 years old.

The prevalence of VL non-suppression varied by selected socio-demographic and socio-behavioural factors. These findings are consistent with other studies that found VL non-suppression varying by age, locality type, educational level, and race.^28,30,31^ The study also found that VL non-suppression was higher among those who reported fair or poor self-rated health. An important observation in our study is that VL non-suppression was significantly higher among adolescents and youth who reported sexual activity in the past 12 months, as observed elsewhere.^32,33,34^ This is particularly worrying when one also looks at low self-report condom use at last sex (38.9%), consistent condom use (28.1%) and condom use among high-risk groups such as black African men (37.5%) and women (35.6%), coupled with rates on multiple sexual partners for males (25.5%) and females (9.0%), particularly among young people.^21,35^ Therefore, when including the high proportion not on ART, 62.9% could transmit HIV.^7^

The final model showed that the odds of VL non-suppression in adolescents and youth aged 12 years – 24 years were age-group specific, more likely among adolescents (15 years – 19 years) and black African people. As observed in other studies, this analysis showed that VL non-suppression was more likely among adolescents.^29,36^ Literature suggests that VL non-suppression among adolescents may be because of ignorance,^30^ non-adherence, which may be attributed to poverty, access (travelling cost and long travelling times and distance), a lack of support (family support), and negative peer pressure because of stigma and health systems barriers among others.^29,36,37,38^ This underlines the need for differentiated services and indicates a need to improve age-appropriate access and support interventions tailored for PLHIV in the population who are being left behind. Such interventions could include strengthening adolescent – and youth-friendly HIV services, and improving family and peer support interventions.^1,39^ In addition, programmes providing care to adolescents should focus on providing routine and intensive adherence counselling to prevent treatment interruptions that can result in VL non-suppression.^18^

### Limitations

The study has several limitations. The behavioural data were self-reported, which introduces reporting bias. However, the survey questions used have been validated over several survey rounds in South Africa. The cross-sectional design cannot establish causality but, only the presence or absence of associations. The small sample size and large CI reduce the statistical power. Another limitation is the fact that the study could not differentiate between those with perinatally acquired HIV and those who acquired it horizontally. The analysis is also limited because of the survey data being over 5 years old, which predates the introduction of dolutegravir (DTG), a new effective and better tolerated antiretroviral agent. Consequently, the findings may not reflect the current landscape as increasing use of DTG should improve treatment outcomes. Another possible limitation is the use of a VL of 1000 copies/mL as a cut-off for undetectable VL. However, evidence shows that it is relevant for resource constrained settings where the use of dried blood sample as in the current survey is more feasible.^40^ Furthermore, a cut-off of 1000 copies/mL has greater utility regarding intervention decision making as it indicates virologic failure when treatment fails to suppress the VL.^41^ This study is based on nationally representative data for a unique age group, the same design can be used to track changes, and/or progress made overtime.

## Conclusion

The study highlights the need for behavioural change interventions as part of ART education and counselling to reduce both the likelihood of VL non-suppression and the risk of HIV transmission especially among adolescent. Additionally, there is a need for age group specific adolescent and youth-friendly healthcare systems and innovative targeting to improve HIV treatment outcomes in this population group. The study also underscores the importance of disclosure, ART initiation and adherence among those with horizontally acquired HIV.

## References

[1] United Nations International Children’s Emergency Fund (UNICEF). HIV treatment, care, and support for adolescents living with HIV in Eastern and Southern Africa. A review for Interventions Scale [homepage on the Internet]. 2021 [cited n.d.]. Available from: https://www.unicef.org/esa/media/8791/file/Adolescents-HIV-Eastern-Southern-Africa-2021.pdf

[2] United Nations International Children’s Emergency Fund (UNICEF). UNICEF data, country profile, South Africa [homepage on the Internet]. 2022 [cited 2024 May 29]. Available from: https://data.unicef.org/country/zaf/#hiv-aids

[3] United Nations Population Fund (UNFPA). One pager on youth demographics [homepage on the Internet]. 2022 [cited 2024 May 29]. Available from: https://www.unfpa.org/sites/default/files/resource-pdf/One%20pager%20on%20youth%20demographics%20GF.pdf

[4] He E, Tolmay J, Zhou S, Saal W, Toska E. Mode of HIV acquisition among adolescents living with HIV in resource-limited settings: A data-driven approach from South Africa. PLoS One. 2023;18(2):e0281298. 10.1371/journal.pone.0281298 36827268 PMC9955664

[5] Pillay T, Cornell M, Fox MP, et al. Recording of HIV viral loads and viral suppression in South African patients receiving antiretroviral treatment: A multicentre cohort study. Antiviral Ther. 2020;25(5):257–266. 10.3851/IMP3371 PMC7982353 32960187

[6] Johnson LF, Patrick M, Stephen C, et al. Steep declines in pediatric AIDS mortality in South Africa, despite poor progress towards pediatric diagnosis and treatment targets. Pediatr Infect Dis J. 2020;39(9):843. 10.1097/INF.0000000000002680 32433224 PMC7958302

[7] Mazanderani AH, Sherman GG. Paediatric and adolescent HIV viral load monitoring, 2014–2020. Nat Inst Commun Dis Bull. 2020;18:165–172.

[8] UNAIDS. Start Free, Stay Free, AIDS Free: 2020 progress report [homepage on the Internet]. Geneva: UNAIDS; 2020 [cited 2024 May 27]. Available from: https://www.unaids.org/sites/default/files/media_asset/2020_global-AIDS-report_en.pdf

[9] UNAIDS. 2020 Global AIDS update – Seizing the moment – Tackling entrenched inequalities to end epidemics [homepage on the Internet]. Geneva: UNAIDS; 2020 [cited 2024 May 29]. Available from: https://www.unaids.org/en/resources/documents/2020/global-aids-report

[10] Cluver L, Orkin M, Campeau L, et al. Improving lives by accelerating the sustainable development goals for adolescents living with HIV: A prospective cohort study. Lancet Child Adolesc Health. 2019;3(4):245–254. 10.1016/S2352-4642(19)30033-1 30878118 PMC6559259

[11] UNAIDS. UNAIDS data 2023. Geneva: Joint United Nations Programme on HIV/AIDS; 2023.

[12] LoVette A, Kuo C, Giovenco D, Hoare J, Operario D. Pre-exposure prophylaxis as an opportunity for engagement in HIV prevention among South African adolescents. SAHARA J. 2022;19(1):1–7. 10.1080/17290376.2021.2016479 35135437 PMC8843204

[13] Bacha JM, Dlamini S, Anabwani F, et al. Achieving antiretroviral therapy uptake and viral suppression among children and adolescents living with HIV in the UNAIDS 90-90-90 era across six countries in Eastern and Southern Africa–lessons from the BIPAI Network. J Acquir Immune Defic Syndr. 2022;90(3):300–308. 10.1097/QAI.0000000000002957 35364599

[14] UNAIDS. Understanding Fast-Track: Accelerating action to end the AIDS epidemic by 2030 [homepage on the Internet]. 2015 [cited 2023 Nov 11]. Available from: unaids.org/sites/default/files/media_asset/201506_JC2743_Understanding_FastTrack_en.pdf

[15] UNAIDS. The path that ends AIDS: UNAIDS Global AIDS update 2023. Geneva: Joint United Nations Programme on HIV/AIDS; 2023.

[16] Joseph Davey D, Abrahams Z, Feinberg M, et al. 2018. Factors associated with recent unsuppressed viral load in HIV-1-infected patients in care on first-line antiretroviral therapy in South Africa. Int J STD AIDS. 29(6):603–610. 10.1177/0956462417748859 29334886 PMC7053422

[17] Mchomvu RD, Hussein AK, Matee M. Determinants of viral load non-suppression among HIV-positive children and adolescents attending care and treatment clinics in Tabora region, Tanzania. Bull Natl Res Centre. 2022;46(1):1–9. 10.1186/s42269-022-00961-3

[18] Nasuuna E, Kigozi J, Babirye L, Muganzi A, Sewankambo NK, Nakanjako D. Low HIV viral suppression rates following the intensive adherence counseling (IAC) program for children and adolescents with viral failure in public health facilities in Uganda. BMC Public Health. 2018;18:1–9. 10.1186/s12889-018-5964-x PMC6103875 30134880

[19] Kruger-Swanepoel GE, Lubbe MS, Rakumakoe DM, Vorster M. Adherence and clinical outcomes of HIV patients switching to a fixed-dose combination regimen. S Afr J Infect Dis. 2022;37(1):464. 10.4102/sajid.v37i1.464 36338195 PMC9634951

[20] Mpolya E. A prospective study of predictors of HIV viral load rebound in an HIV hyperendemic rural population of KwaZulu-Natal, South Africa. PAMJ One Health. Nairobi: Pan Afr. Med. J. 2023;11:13. Available from: 10.11604/pamj-oh.2023.11.13.40381

[21] Tomita A, Vandormael A, Bärnighausen T, et al. Sociobehavioral and community predictors of unsuppressed HIV viral load: Multilevel results from a hyperendemic rural South African population. AIDS (London, England). 2019;33(3):559. 10.1097/QAD.0000000000002100 30702520 PMC6547375

[22] Simbayi LC, Zuma K, Zungu, et al. South African national HIV prevalence, incidence, behaviour and communication survey, 2017. Towards achieving the UNAIDS 90-90-90 targets. Cape Town: Human Sciences Research Council Press; 2019.

[23] Statistics South Africa (StatsSA) census in brief [homepage on the Internet]. 2001 [cited n.d.]. Available from: http://www.statssa.gov.za/census/census_2001/census_in_brief/CIB2001.pdf

[24] Statistics South Africa (StatsSA). Annual report 2011/2012 [homepage on the Internet]. 2011. Available from: https://www.gov.za/sites/default/files/gcis_document/201409/statssa-annualreportjanuary2012reduced.pdf

[25] Hladik W, Stupp P, McCracken SD, et al. The epidemiology of HIV population viral load in twelve sub-Saharan African countries. PLoS One, Public Library of Science. 2023;18(6):e0275560. 10.1371/journal.pone.0275560 PMC10292693 37363921

[26] UNAIDS. Public health and HIV viral load suppression [homepage on the Internet]. Undetectable = untransmittable. 2024. Available from: https://www.unaids.org/sites/default/files/media_asset/undetectable-untransmittable_en.pdf

[27] Mhlanga TT, Jacobs BK, Decroo T, et al. Virological outcomes and risk factors for non-suppression for routine and repeat viral load testing after enhanced adherence counselling during viral load testing scale-up in Zimbabwe: Analytic cross-sectional study using laboratory data from 2014 to 2018. AIDS Res Ther. 2022;19(1):34. 10.1186/s12981-022-00458-z 35810317 PMC9270749

[28] Nyongesa MK, Mwatasa MH, Kagonya VA, et al. HIV virological non-suppression is highly prevalent among 18-to 24-year-old youths on antiretroviral therapy at the Kenyan coast. BMC Infect Dis. 2022;22(1):1. 10.1186/s12879-022-07428-w 35545757 PMC9092782

[29] Umar E, Levy JA, Bailey RC, et al. Virological non-suppression and its correlates among adolescents and young people living with HIV in Southern Malawi. AIDS Behav. 2019;23:513–522. 10.1007/s10461-018-2255-6 30132172 PMC8896833

[30] Sunkanmi F, Paul Y, Peter D, et al. Factors influencing viral load non-suppression among people living with HIV (PLHIV) in Borno State, Nigeria: A case of Umaru Shehu Ultra-Modern Hospital. J Adv Med Med Res. 2020;32(3):98–105. 10.9734/jammr/2020/v32i330388

[31] Blank AE, Fletcher J, Verdecias N, et al. Factors associated with retention and viral suppression among a cohort of HIV+ women of color. AIDS Patient Care STD. 2015;29(S1):S27–S35. 10.1089/apc.2014.0272 PMC4283067 25458205

[32] Okonji EF, Van Wyk B, Mukumbang FC, Hughes GD. Determinants of viral suppression among adolescents on antiretroviral treatment in Ehlanzeni district, South Africa: A cross-sectional analysis. AIDS Res Ther. 2021;18:1–9. 10.1186/s12981-021-00391-7 34627300 PMC8501534

[33] Shisana O, Rehle T, Simbayi LC, et al. South African national HIV prevalence, incidence and behaviour survey, 2012. Cape Town: Human Science Research Council Press; 2014.

[34] Nyakato P, Schomaker M, Fatti G, et al. Virologic non-suppression and early loss to follow up among pregnant and non-pregnant adolescents aged 15–19 years initiating antiretroviral therapy in South Africa: A retrospective cohort study. J Int AIDS Soc. 2022;25(1):e25870. 10.1002/jia2.25870 35032096 PMC8760609

[35] Mthembu Z, Maharaj P, Rademeyer S. ‘I am aware of the risks, I am not changing my behaviour’: Risky sexual behaviour of university students in a high-HIV context. Afr J AIDS Res. 2019;18(3):244–253. 10.2989/16085906.2019.1655075 31575340

[36] Elishi BA, Van Wyk BE. Factors associated with viral suppression among adolescents on antiretroviral therapy in Free State province, South Africa. South Afr J HIV Med. 2022;23(1):1356. 10.4102/sajhivmed.v23i1.1356 35923610 PMC9257832

[37] Hlophe LD, Tamuzi JL, Shumba CS, Nyasulu PS. Barriers and facilitators to anti-retroviral therapy adherence among adolescents aged 10 to 19 years living with HIV in sub-Saharan Africa: A mixed-methods systematic review and meta-analysis. PLoS One. 2023;18(5):e0276411. 10.1371/journal.pone.0276411 37200399 PMC10194875

[38] Zungu N, Naidoo I, Hodes R, et al. Adolescents living with HIV in South Africa. Pretoria: Human Sciences Research Council Press, 2021.

[39] World Health Organization (WHO). The role of HIV viral suppression in improving individual health and reducing transmission [homepage on the Internet]. Policy Brief. 2023 [cited 2023 Nov 11]. Available from: https://www.who.int/publications/i/item/9789240055179

[40] Broyles LN, Luo R, Boeras D, Vojnov L. The risk of sexual transmission of HIV in individuals with low-level HIV viraemia: A systematic review. Lancet. 2023;402(10400):464–471. 10.1016/S0140-6736(23)00877-2 37490935 PMC10415671

[41] Harlow AF, Bor J, Brennan AT, et al. Impact of viral load monitoring on retention and viral suppression: A regression discontinuity analysis of South Africa’s National laboratory cohort. Am J Epidemiol. 2020;189(12):1492–1501. 10.1093/aje/kwaa140 32648905 PMC7705603

